# Pathophysiology and medical treatment of pain in fibrous dysplasia of bone

**DOI:** 10.1186/1750-1172-7-S1-S3

**Published:** 2012-05-24

**Authors:** Roland D Chapurlat, Deborah Gensburger, Juan M Jimenez-Andrade, Joseph R Ghilardi, Marilyn Kelly, Patrick Mantyh

**Affiliations:** 1 INSERM UMR 1033, Université de Lyon, Hospices Civils de Lyon, Hôpital E Herriot, 69437 Lyon, France; 2 National Reference Center for Fibrous Dysplasia of Bone, Hôpital E Herriot, 69437 Lyon, France; 3 Department of Pharmacology, College of Medicine, University of Arizona, Tucson, AZ 85724, USA; 4 Research Service, VA Medical Center, Minneapolis, MN 55417, USA; 5 Skeletal Clinical Studies Unit, Craniofacial and Skeletal Diseases Branch, National Institute of Dental and Craniofacial Research, National Institutes of Health, Bethesda, MD, USA; 6 Arizona Cancer Center, University of Arizona, Tucson, AZ 85724, USA

## Abstract

One of the most common complications of fibrous dysplasia of bone (FD) is bone pain. Usual pain killers are often of inadequate efficacy to control this bone pain. The mechanism of bone pain in FD remains uncertain, but by analogy with bone tumors one may consider that ectopic sprouting and formation of neuroma-like structures by sensory and sympathetic nerve fibers also occur in the dysplastic skeleton. Bone pain has been reported in up to 81% of adults and 49% of children. It affects predominantly the lower limbs and the spine. The degree of pain is highly variable and adults reports more pain than children. Bisphosphonates have been shown to reduce bone pain in uncontrolled studies. Their influence on bone strength remains unknown. In a randomized trial testing alendronate, bone pain was not significantly improved. Another trial assessing the effect of risedronate is ongoing. Possible future therapies include tocilizumab, denosumab and drugs targeting nerve growth factor and its receptor TrkA.

## Introduction

Fibrous dysplasia of bone (FD) is a rare disease responsible for bone deformities, fractures, nerve compression and bone pain. There are specificities in the pathophysiology of bone pain compared to other tissues, including the role of increased bone resorption. The treatment of bone pain can involve non specific drugs and bone-specific drugs, such as bisphosphonates.

We will review the pathophysiology of bone pain, the current therapeutic possibilities and the treatment perspectives.

## Pathophysiology of bone pain

Pain is a common occurrence in FD and is often the presenting symptom of the disease [1-3]. When the health-related quality of life was assessed in FD subjects, both adults and children had significantly more skeletal pain than the U. S. population [4]. A common misconception is that FD pain dissipates with age; however, recent population studies suggest that FD pain actually increases with age [3]. The analgesics that are most commonly used to control FD pain are non-steroidal anti-inflammatory drugs (NSAIDS), bisphosphonates and opiates [2,3]. However, lack of recognition by the medical community that FD pain can be both severe and increase in adulthood has led many FD patients to be labeled as “drug seeking” and inadequately treated [3]. Adequate pain management of FD pain, like nearly all other types of pain, is clearly required for FD patients to maintain their functional status and quality of life.

Currently, our understanding of the factors that drive FD pain and how to best treat FD pain comes mainly from empirical studies concerning the ability of available therapies to relieve FD pain. Two seminal clinical studies included one where it was demonstrated that FD pain was attenuated following infusion of the bisphosphonate pamidronate [5]. The second showed that there was not a clear correlation between FD pain and disease burden, and that in terms of frequency and severity FD pain increases with age [3]. This later finding may in part be explained by the fact that whereas bone mass, density, and strength all decline with age, sensory nerve fibers that innervate bone and which sense noxious stimuli and transmit this information to the spinal cord and brain, do not appear to decline with age [6].

While there are currently no direct studies examining what mechanisms drive FD pain, in the last decade significant strides have begun to be made in understanding the specific populations of sensory nerve fibers that innervate the skeleton [7,8], what mechanisms drive malignant and non-malignant skeletal pain [9], what molecules preferentially excite nerve fibers that innervate the bone [9], and what analgesic therapies may be particularly efficacious in alleviating skeletal pain [10].

## A select population of sensory nerve fibers innervates the skeleton and drives skeletal pain

**Figure 1:**
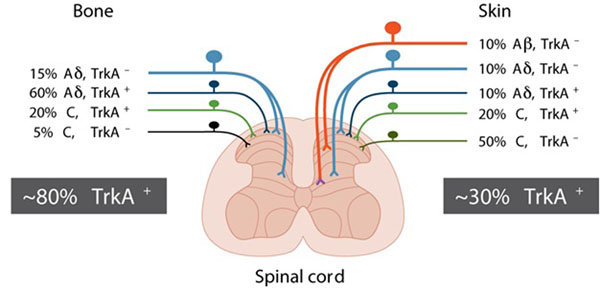
**Most sensory nerve fibers that innervate the bone express TrkA whereas fewer than 30% of the nerve fibers that innervate the skin express TrkA.** The skin is innervated by thickly myelinated A-beta fibers (TrkA^-^), thinly myelinated A delta fibers (both TkA^-^ and TrkA^+^), unmyelinated peptide-rich C fibers (TrkA^+^) and unmyelinated peptide-poor C-fibers (TrkA^-^). In contrast, the bone appears to be predominantly innervated by thinly myelinated A-delta fibers (TrkA^-^ but mostly TrkA^+^) and peptide-rich C-fibers (mostly TrkA^+^ and a small proportion TrkA^-^). As greater than 80% of all sensory nerve fibers that innervate the bone are TrkA^+^ whereas only 30% of the sensory nerve fibers that innervate skin are TrkA^+^, these data might help explain why blocking NGF or its cognate receptor TrkA appears to be more efficacious in attenuating skeletal vs. skin pain.

Bone is primarily innervated by thinly myelinated sensory nerve fibers (A-delta) and peptide-rich CGRP^+^ nerve fibers and thus has less “redundancy” than is found in skin. These nerve fibers may express the high affinity nerve growth factor (NGF) receptor, Trk A, which mediates the multiple effects of NGF, including neuronal differentiation and survival. That pattern of innervation is present in the periosteum, mineralized bone, and marrow [7,8] (Figure 1). These results suggest that this differential population may provide a unique therapeutic opportunity for developing novel analgesics that can attenuate FD skeletal pain as fewer populations of nerve fibers will be needed to be blocked to attenuate bone compared to skin pain.

## Sensory nerve fibers that innervate the skeleton can undergo a remarkable sprouting and pathological reorganization which may drive FD pain

**Figure 2:**
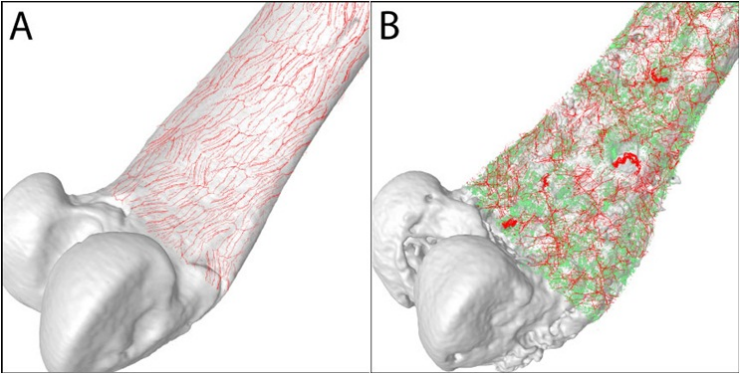
**Sprouting and formation of neuroma-like structures in chronic bone pain.** Sarcoma tumor cells expressing green fluorescent protein (green) induce a marked sprouting and neuroma formation of CGRP^+^ sensory nerve fibers (red) that innervate the bone (white). These nerve fibers detect and transmit painful stimuli from periphery to the central nervous system. **A)** In sham bones, CGRP^+^ nerve fibers that are present in the bone appear as single nerve fibers with a liner and homogenous morphology. **B)** As cancer cells proliferate and grow in bone, these induce significant bone remodeling (pitted appearance) as well as a highly pathological sprouting and formation of neuroma-like structures by sensory and sympathetic nerve fibers which in other conditions drives chronic pain. Confocal images from periosteal whole preparations were acquired and overlapped on a three dimensional image of the mouse femur obtained by microcomputed tomography. Images were rendered courtesy of Marvin Landis (University Information Technology Services, University of Arizona).

One possible explanation as to why there is not a direct correlation between disease burden and FD pain is that it is not bone remodeling alone that drives bone pain, but that sensory nerve fibers themselves also have to undergo a pathological change. Recently, it has been shown that when osteosarcoma cells are confined and grow within the bone, there is a remarkable and ectopic sprouting and formation of neuroma-like structures by sensory and sympathetic nerve fibers in the skeleton (Fig. 2). Interestingly, sustained administration of an anti-NGF sequestering therapy blocked the pathological sprouting of sensory and sympathetic nerve fibers, the formation of neuroma-like structures, and significantly attenuated the generation and maintenance of cancer pain in this model [11].

**Figure 3:**
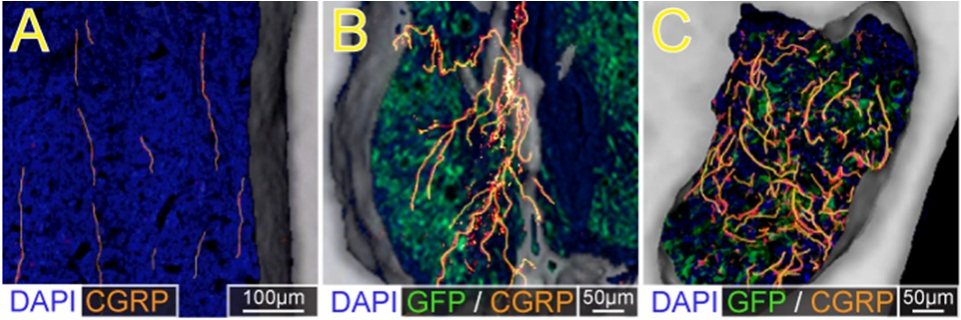
**Prostate cancer cells induce sprouting of sensory nerve fibers in the bone marrow of tumor bearing femurs.** High power µCT slices of bone (100 µm-thick) overlaid with confocal images (20 µm-thick) obtained from a sham femur (**A**) and tumor-bearing femur from mice sacrificed at early (**B**) and more advanced stages of the disease (**C**). In these images the DAPI stained nucleus of cells appear blue, the green fluorescent protein expressing (GFP) prostate cancer cells appear green, and the calcitonin gene related peptide (CGRP) sensory nerve fibers appear yellow/red. Note that in the sham mice, CGRP^+^ nerve fibers that are present in the marrow space of normal mice appear as single, nerve fibers with a highly linear morphology. As GFP^+^ prostate tumor cells proliferate and form tumor colonies (**B,C**), the CGRP^+^ sensory nerve fibers undergo marked sprouting which produces highly branched, disorganized and dense meshwork of sensory nerve fibers (**B,C**) that is never observed in the normal marrow (**A**).

A major question is whether this ectopic sprouting of sensory nerve fibers only occurs when cancer cells express high levels of NGF. However, studies using canine prostate cancer cells, that do not express detectable levels of NGF [12] – as is observed in FD – simultaneously induce excessive bone growth and pathological bone remodeling (Fig. 3). A similar ectopic sprouting of sensory and sympathetic nerve fibers occurs in the bone marrow and mineralized bone [13]. As these prostate cells do not express detectable levels of mRNA coding for NGF, these data suggest that this ectopic sprouting of nerve fibers is not primarily driven by NGF released from tumor cells, but rather by the major source of NGF arising from endogenous stromal, inflammatory and immune cells [14,15]. These newly sprouted nerve fibers are probably also activated and sensitized by released NGF and as such this truly ectopic and pathological reorganization of sensory and sympathetic nerve fibers may provide an anatomical substrate which drives skeletal pain. In support of this hypothesis, preventive treatment with an antibody that sequesters NGF, administered when prostate tumor-induced pain and bone remodeling were first observed, blocked this ectopic sprouting and significantly inhibited the development and severity of cancer pain [13].

While it is not known whether sprouting of sensory nerve fibers occurs in FD, this phenomenon has been observed in non-malignant skeletal pain states in human and animals. Previous studies have reported that human chronic discogenic pain may in part be due to a growth of TrkA^+^ nerve fibers into normally aneural and avascular areas of the human intervertebral disc [16]. Other studies have demonstrated significant sprouting of CGRP^+^ nerve fibers following bone fracture in rat and in the arthritic joints of humans and animals [17-19]. These reports suggest that following injury or disease of the skeleton, significant sprouting of TrkA^+^ nerve fibers can occur, and it appears that endogenous stromal cells as well as inflammatory and immune cells are the source of NGF [14,15].

## The burden of bone pain in fibrous dysplasia

### Methods and patients (adapted from Ref. 4)

We have studied a relatively large population of patients with FD in an effort to understand their experience with pain [4]. All subjects enrolled in a National Institutes of Health (NIH) Institutional Review Board approved study of FD and MAS were invited to complete the self report Brief Pain Inventory (BPI) and a demographic data questionnaire during their initial evaluation at NIH between July 2000 and July 2005. Ninety-one subjects were enrolled during that period, and 78 (86%) completed the pain form and had a ^99^Tc-MDP bone scan, including 56 subjects 14 or older (72%) and 22 under the age of 14 (28%). The diagnosis of FD was established in all patients based on clinical history, histopathological findings, radiographic findings, and when necessary, an analysis of the *GNAS* gene for R201 mutations. Bone scans were assessed for sites of FD involvement, which were identified as areas of non-physiologic tracer uptake, and disease severity was determined using a validated scoring tool [20]. The fact that tracer uptake sites represented FD was confirmed by radiograph and/or CT. Pain was assessed using a human figure drawing and the numeric rating scale (NRS) of the Brief Pain Inventory (BPI). The BPI is a short, self-administered questionnaire developed to assess the severity and impact of pain primarily in cancer patients [21]. It has been shown to be valid and reliable in adults when used to assess cancer pain [21], chronic and acute nonmalignant pain and pain in osteoarthritis patients. The goal was to assess pain “intrinsic” to the FD and not pain that occurred in relation to a fracture. Therefore, acute or healing fractures were excluded from the analysis (i.e., > 6 months since radiographic evidence of complete healing at a site at which there had been a recent fracture). Analgesic use and perceived relief information was obtained as part of the questionnaire, and confirmed during patient interviews.

## Results

The study population was made up of a group of subjects with a broad spectrum of disease, from isolated monostotic FD, to total skeletal involvement. The lower extremities were the sites most likely to be affected by FD (86% of adults, 97% of children, p=NS for differences between adults and children). The head was also commonly affected (86% of adults, 94% of children, p=NS). FD lesions were found less frequently in the upper extremities (72% of adults, 89% of children, p=NS), the ribs (72% of adults, 57% of children, p=NS) and the spine (72% of adults, 46% of children, p<0.05). The spine was the only site at which there was a significant increase in FD involvement over time.

**Figure 4:**
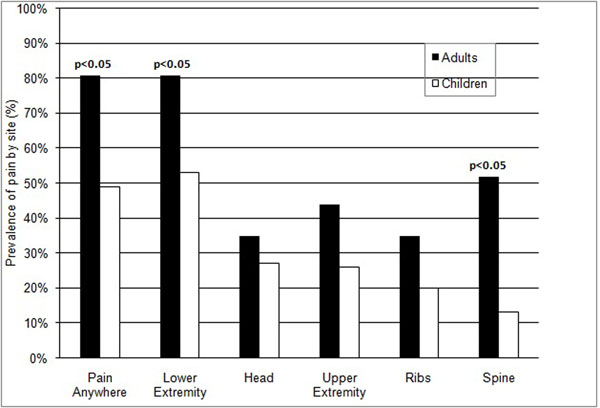
**Prevalence of pain at skeletal sites involved with fibrous dysplasia in adults and children.** Sites at which patients reported pain was recorded. 99Tc-MDP bone scans were reviewed to confirm the presence of FD at the reported site of pain. Only those sites at which there was a concordance of pain and FD involvement were recorded. Adults had significantly more pain than children in general (p<0.05), and at both the lower extremity and the spine (p<0.05 for both).

Pain was prevalent in the FD population; 67% reported pain at FD sites. Pain was more common in adults than children, and was reported by 81% of adults and 49% of children (p<0.005) (Fig. 4). Adults reported significantly more pain than children in both the lower extremities (adults 81%, children 53%, p<0.05) and the spine (adults 52%, children 13%, p<0.05) (Figure 2). The degree of pain reported was considerable, but quite variable. The mean pain score (on the 0 to 10 pain scale) for adults was 4.1 (range 1 to 8, ± 1.8), and 2.8 for children (range 1 to 7, ± 1.8) (Table 1). Adults had significantly more pain than children (p<0.01). In an effort to assess how pain prevalence changed with aging, we examined the prevalence of pain in age group increments of 10 years. No pain was reported by children less than 10 years old, while 50-60% of those age 11 through 30 reported pain and 85-100% of the patients over 31 years of age experienced pain (Table 1).

**Table 1:** Prevalence of pain by age groups

Age group (years)	n	% of subjects with pain
<10	7	0
11-20	27	59
21-30	10	50
31-40	13	85
41-50	14	100
>50	7	86

There was no correlation between pain prevalence and gender, phosphate wasting, vitamin D status (serum vitamin D level < 32 ng/ml was used as a cutoff for the diagnosis of vitamin D deficiency), or any endocrinopathy in children. Growth hormone excess correlated with pain prevalence at FD sites in adults (p=0.031).

Patients reported using a variety of treatments to control pain (Table 2). NSAIDs were most commonly used (57% of adults and 56% of children who had pain). Some subjects reported using more than one treatment. There was a trend for children who reported pain to be less likely to be treated for pain than adults (p=0.21).

**Table 2:** Pain severity, treatment and response to treatment^1^

	Adults		Children	
**Average pain**	4.1*		2.8	
**Treatment**	% treated	% with releif	% treated	% with releif
**No treatment**	26%		44%	
**NSAIDs**	57%	56%	56%	50%
**Narcotics**	26%	47%	17%	90%
**Bisphosphonates**	26%	73%	17%	75%
**Alternative Treatments**	17%	52%	11%	No report

^1^Only subjects who had FD-associated pain are recoded in this analysis.

*=p<0.05, NSAIDs = non steroidal anti-inflammatory drugs

Figure 4 and Tables 1 and 2 reprinted with permission from the National Osteoporosis Foundation, Washington, DC 20036. Osteoporosis Int (2008) 19:57-63: All rights reserved.

## Treatment of fibrous dysplasia bone pain with bisphosphonates

The use of an antiresorptive agent in the treatment of an osteoblastic lineage disease, such as FD, is counterintuitive. The rationale for doing so is based on the presence of abundant osteoclastic bone resorption within and around the fibrous tissue. Therefore, in an early study that took as an example the treatment of Paget’s disease, 9 patients were treated with intravenous pamidronate (180 mg every 6 months), with striking radiographic improvements and decreases in bone pain and biochemical markers of bone remodeling [5]. Patients were also receiving calcium (500-1500 mg/day) and vitamin D (800-1200 IU/day) supplements.

Long-term effects of this regimen have been assessed with additional patients and longer follow-up, still in an open design, with similar results [2,22]. A dose of 3 mg/kg/treatment cycle was used in children and adolescents, who represented 30% of this cohort. Fifty-eight patients have been treated with intravenous pamidronate and followed-up for an average 50 months (ranging from 1 to 11 years). Pain intensity was reduced after the first course of treatment, with an additive effect observed after several treatment cycles. Bone pain disappeared in 60% of these patients, diminished in 24% and did not improve in 16% of them. In parallel, biochemical markers of bone turnover – such as total alkaline phosphatase, serum osteocalcin, and urinary CTX – were also significantly reduced compared to baseline. Half of those treated patients had discernable radiological improvement, characterized by filling of osteolytic lesions and/or cortical thickening. In addition, total hip bone mineral density (BMD) measured in patients who had hip involvement was substantially increased [23]. Results were similar in adults and children or adolescents. These biochemical and radiological changes, however, were not associated with bone pain reduction.

Favorable outcomes have also been observed in other open studies using intravenous pamidronate, administered at 6-month intervals. Thus, bone pain was significantly relieved in a study involving 7 patients with various forms of FD treated with intravenous pamidronate [24]. A greater increase in BMD was also observed in affected areas than in unaffected areas, using whole body DXA to compare the affected to the unaffected side, after 1 year of treatment. Simultaneously, the level of bone turnover as assessed by biochemical markers was reduced but most patients still had increased bone turnover.

A few patients have also been treated successfully with alendronate. For example, an increase of 158% in total hip BMD over 2 years has been observed in a 22-year old woman who had received four 90 mg infusions of pamidronate every 4 weeks, followed by oral alendronate 10 mg/day [25], with a parallel relief in bone pain and decrease in urinary NTX. In another case report [26], a 45-year old woman who received alendronate 5 mg/day was relieved of her bone pain after several months of treatment. Bone turnover was diminished and the radiological appearance improved slightly. In a series of 6 adult patients who had been treated with pamidronate followed by alendronate or who had used alendronate alone, bone pain decreased substantially in response to therapy, bone resorption was reduced with intravenous pamidronate but not with oral alendronate, and four out of six patients exhibited radiological improvement [27].

Although most patients respond favorably to pamidronate therapy, a subset (15% in our group's experience, RDC) did not exhibit any improvement in bone pain. Other patients, with an initial positive response to treatment with pamidronate, have suffered from a relapse of bone pain or failed to maintain reduced levels of biochemical markers of bone turnover. When those patients who relapsed or failed treatment with pamidronate were switched to zoledronic acid, we were not able to obtain significant reductions in bone pain or improvement in the radiographic appearance [28]. Zoledronic acid was well-tolerated, with only two patients with an acute phase reaction associated with the first infusion. Those patients switched to zoledronic acid tended to have more serious disease than the other patients on pamidronate only.

In another study [29], however, no convincing evidence of radiographic benefit could be observed in 18 children and adolescents with polyostotic FD, despite significant reduction in levels of bone turnover markers. The explanation for the discrepancy between this study conducted in young patients and those in adults or other pediatric series [30-32] remains unclear, but some of the difference might relate to the absence of use of phosphate supplements in those patients with renal phosphate wasting. The difference may also stem from the difficulty in defining appropriate radiographic outcomes in studies of FD, as lesions are heterogeneous and radiographs are not always reproducible over time.

All these results were obtained in uncontrolled open studies. The role of the placebo effect and regression to the mean is likely to explain some of the effect on bone pain. The radiologic effect might be confounded by the age-related sclerosis of lesions [33], but this phenomenon arises over long periods of time, whereas the improvement associated with bisphosphonate use could be observed over shorter periods of time, e.g., 2-3 years. These shortcomings led to the design of two randomized placebo-controlled clinical trials, one conducted in the USA to test alendronate [34], and the other in Europe, the PROFIDYS trial, testing risedronate [35]. The results of the first trial are not yet published, and the latter is still recruiting patients.

## Perspectives

Some patients fail to respond to bisphosphonates or relapse after an initial improvement in bone pain. These individuals do not seem to respond better to more potent bisphosphonates, such as zoledronic acid [28]. Those patients often have severe polyostotic disease, with a history of several fractures, substantial bone pain, and sometimes optic nerve compression. There is no current satisfactory therapeutic option in these severely disabled patients whose disease is resistant to bisphosphonates.

We know that *GNAS* mutations result in abnormal proliferation and differentiation of bone marrow stromal cells. In those osteoblastic cells, IL-6 secretion is increased as a result of Gs activation, with consequent activation of surrounding osteoclasts, allowing the FD lesion to expand and create osteolytic lesions [36]. A direct link has been established between the *GNAS* mutation in stromal cells and IL-6 production, so that FD, which is an osteoblastic lineage disorder, is also often associated with a hyperosteoclastic component [37].

This is the rationale to selectively inhibit the IL-6 driven increased bone resorption that is observed in FD by targeting the IL-6 receptor with tocilizumab, in those patients who fail to respond to bisphosphonates primarily or secondarily. Tocilizumab - a human monoclonal antibody to IL-6 receptor - is a drug currently used in rheumatoid arthritis (RA) treatment. It can reduce symptoms, and block localized periarticular bone loss induced by the disease. A recent study has also shown that the level of systemic bone resorption, as assessed by markers such as serum ICTP and CTX could be significantly decreased in RA in response to tocilizumab [38].

Therefore, a randomized placebo-controlled cross-over trial testing the value of tocilizumab to decrease bone resorption among patients with FD who do not respond to bisphosphonate therapy will be launched in Europe in 2011. A total of 12 patients will receive either tocilizumab during 6 months followed by 6 months of placebo (6 patients), or 6 months of placebo followed by 6 months of tocilizumab (6 patients). The study is powered to show a 30% difference in bone resorption between the two treatments. Decrease in bone resorption (primary endpoint) will be assessed with serum CTX. Secondary endpoints will be: decrease in bone pain, assessed by visual analogic scale in the most painful skeletal site, decrease in other markers of bone remodeling (serum osteocalcin, bone alkaline phosphatase, P1NP), and improvement in the short-form 36 (SF-36) quality of life scale.

Another way to develop new therapies to treat pain associated with fibrous dysplasia is to understand the unique populations of nerve fibers that innervate bone and the mechanisms by which these nerve fibers signal skeletal pain. Unlike skin, the majority of sensory nerve fibers in bone express TrkA, TRPV1 antagonists, inhibitors of CSFR1 and pregabalin (Table 3). In addition to assessing the efficacy of these therapies to reduce FD pain, endpoints which need to be included in these clinical trials are effect on disease progression, side effect profile and risk/benefit to the patient. Additionally, developing an animal model of FD and understanding how the density, morphology, phenotype, and response characteristics of skeletal sensory nerve fibers changes in a pre-clinical model of FD may help in the development of more targeted therapies to treat FD pain.

**Table 3:** Therapies that may be useful in treating FD pain

CURRENT AND POTENTIAL THERAPIES FOR TREATMENT OF FIBROUS DYSPLASIA PAIN			
**DRUG CLASS**	**TARGET**	**ACTION**	**POTENTIAL COMPLICATIONS**

**Current therapies**			
Biphosphonates	Osteoclasts	Osteoclast apoptosis	Inhibition of bone remodeling/
		Osteoclast activity suppression	growth Osteonecrosis
Opioids	CNS neurons	Stimulates opioid receptors	Sedation Dependence Constipation
NSAIDS	Prostaglandin synthesis	Blockade of peripheral and central sensitization	GI toxicity Cardiotoxicity Nephrotoxicity

**Recently approved therapies/ ongoing clinical trials for treating other skeletal pain states**			

Denosumab (OPG)	Blocks RANKL	Blocks osteoclast activation	Inhibition of bone remodeling/
(Amgen)			growth Osteonecrosis
Tanezumab (anti-NGF) (Pfizer)	NGF/TrkA pathway	Blockade of peripheral sensitization Blockade of nerve sprouting	Developing sensory and sympathetic nerve fibers

**Potential therapies**			

NGF/TrkA inhibitors (Array, JNJ, Abbott)	NGF/TrkA pathway	Blockade of peripheral sensitization Blockade of pH sensitive neurons	Developing sensory and sympathetic nerve fibers
TRPV1 antagonists (Pfizer, JNJ, Abbott, Merck, GSK, etc.)	TRPV1 channel	Blockade of pH sensitive neurons	Hyperthermia (transient?)
CSFR1 inhibitors response (Plexxikon, Roche, JNJ)	Inhibition of CSFR1	Reduction in osteoclasts, macrophages, etc.	Decreased immune response to infection
Pregabalin (Pfizer)	Calcium channel, α2, δ1 subunit	Aberrant neuronal discharge	Lethargy Drowsiness

Denosumab is currently approved to treat osteoporosis as it targets RANKL and is remarkably effective at reducing osteoclast-induced bone remodeling. As such it may represent a potential treatment for FD bone pain. Pregabalin has been shown to attenuate a wide variety of neuropathic pain (i.e. pain originating from damaged or ectopic reorganization of nerve fibers) and as ectopic reorganization of nerve fibers may play an important role in driving FD induced skeletal pain, pregabalin might be useful in reducing FD pain, however no clinical study data currently exists that specifically investigates the efficacy of pregabalin in FD.

## Conclusion

Bone pain is commonly observed in FD. Bisphosphonates can provide some relief of bone pain, but the development of mechanism-based therapies to treat neuropathic bone pain or the bone disease itself is needed to improve the management of FD patients.

## Competing interests

The authors declare that they have no competing interests.
